# Environmental enrichment intervention for Rett syndrome: an individually randomised stepped wedge trial

**DOI:** 10.1186/s13023-017-0752-8

**Published:** 2018-01-10

**Authors:** Jenny Downs, Jenny Rodger, Chen Li, Xuesong Tan, Nan Hu, Kingsley Wong, Nicholas de Klerk, Helen Leonard

**Affiliations:** 1 Telethon Kids Institute, The University of Western Australia, PO Box 855, West Perth, WA 6872 Australia; 2 School of Physiotherapy and Exercise Science, Curtin University, Perth, WA Australia; 3 School of Biological Sciences, The University of Western Australia, WA, Perth, Australia; 4 Department of Neurology, Shenzhen Children’s Hospital, Shenzhen, China; 5 Rett Syndrome Comprehensive Research Institute, Shenzhen, China

**Keywords:** Rett syndrome, Neurodevelopmental disorder, Environmental enrichment, Neuroplasticity, BDNF

## Abstract

**Background:**

Rett syndrome is caused by a pathogenic mutation in the *MECP2* gene with major consequences for motor and cognitive development. One of the effects of impaired *MECP2* function is reduced production of Brain Derived Neurotrophic Factor (BDNF), a protein required for normal neuronal development. When housed in an enriched environment, MECP2 null mice improved motor abilities and increased levels of BDNF in the brain. We investigated the effects of environmental enrichment on gross motor skills and blood BDNF levels in girls with Rett syndrome.

**Methods:**

A genetically variable group of 12 girls with a *MECP2* mutation and younger than 6 years participated in a modified individually randomised stepped wedge design study. Assessments were conducted on five occasions, two during the baseline period and three during the intervention period. Gross motor function was assessed using the Rett Syndrome Gross Motor Scale (maximum score of 45) on five occasions, two during the baseline period and three during the intervention period. Blood levels of BDNF were measured at the two baseline assessments and at the end of the intervention period. The intervention comprised motor learning and exercise supplemented with social, cognitive and other sensory experiences over a six-month period.

**Results:**

At the first assessment, the mean (SD) age of the children was 3 years (1 year 1 month) years ranging from 1 year 6 months to 5 years 2 months. Also at baseline, mean (SD) gross motor scores and blood BDNF levels were 22.7/45 (9.6) and 165.0 (28.8) ng/ml respectively. Adjusting for covariates, the enriched environment was associated with improved gross motor skills (coefficient 8.2, 95%CI 5.1, 11.2) and a 321.4 ng/ml (95%CI 272.0, 370.8) increase in blood BDNF levels after 6 months of treatment. Growth, sleep quality and mood were unaffected.

**Conclusions:**

Behavioural interventions such as environmental enrichment can reduce the functional deficit in Rett syndrome, contributing to the evidence-base for management and further understanding of epigenetic mechanisms. Environmental enrichment will be an important adjunct in the evaluation of new drug therapies that use BDNF pathways because of implications for the strengthening of synapses and improved functioning.

**Trial registration:**

ACTRN12615001286538.

## Background

Rett syndrome is caused by a pathogenic variant in the *MECP2* gene [1] resulting in major consequences for the development of motor functioning and cognitive skills. Motor effects include loss of hand function skills during a regression period [2] with a slow decline in gross motor skills over time [3]. Environmental enrichment models are components of most early intervention programs for children with a neurodevelopmental disability evaluated most frequently in cerebral palsy [4]. Although similar programs also exist for Rett syndrome, their evidence base is poor [5]. In a single subject design study with three affected girls, participation in conductive education improved gross motor skills [5].

One of the effects of impaired *MECP2* function is reduced production of Brain Derived Neurotrophic Factor (BDNF), a protein required for normal neuronal development and brain function [6]. There is some clinical evidence of a role for *BDNF* in Rett syndrome pathogenesis [7–9]. Relationships between the type of *BDNF* polymorphism and phenotype have been demonstrated, with more clinical severity and earlier seizure onset when limited to the p.Arg168* mutation [7]. Overall, the age of seizure onset has been observed as earlier [9] or slightly later [7] in individuals with the heterozygous (Val/Met) irrespective of mutation type. In another study, no child with a heterozygous (Val/Met) *BDNF* polymorphism developed epilepsy earlier than 2 years [8]. Environmental enrichment reduces cellular and behavioural deficits in animal models of disorders including Huntington’s disease, Fragile X and Down syndrome [10], and clinical improvements have been found in Parkinson’s and Alzheimer’s diseases [11] and cerebral palsy [4]. When housed in an enriched environment, *MECP2* null mice have demonstrated improved motor abilities and increased levels of BDNF in the brain [12]. Environmental enrichment could benefit children in the early stages of Rett syndrome and increased BDNF levels could be associated with improvements. Environmental enrichment could effect wellbeing more generally. For example, difficulties getting to sleep and staying asleep [13] occur frequently in Rett syndrome [14]. Exercise could improve these symptoms and potentially improve behavioural outcomes such as mood [15].

Based on the incidence of Rett syndrome in Australia [16], approximately 1000 girls of the 8.6 million girls born each year in China and approximately 222 of the nearly 2 million girls born each year in the US will have Rett syndrome. Whilst Rett syndrome is rare, many girls are affected globally. This study examined the effects of environmental enrichment on gross motor skills and BDNF protein levels in Rett syndrome. As secondary outcomes, sleep quality and mood were also evaluated.

## Methods

### Participants and setting

The international database InterRett established in 2002 [17] was used for recruitment to this study. Families with a child with a confirmed clinical diagnosis of Rett syndrome [18] aged two to 6 years were invited to participate, provided their child also had a pathogenic *MECP2* mutation. Assessments and intervention were conducted at the Rett Syndrome Comprehensive Research Institute (RSCRI) in Shenzhen, China in 2016.

### Trial design

A modified stepped wedge individually randomized controlled trial [19] was conducted with different girls crossing over from control to intervention conditions at different time points. The baseline period was either one, two or 3 months as determined randomly and thereafter the intervention period was 6 months. The stepped wedge design was chosen because 1) variability between genotypes limited our capacity to randomize into two similar groups within the available sample [20, 21] and 2) it was considered reasonable and standard practice for the girls to have to wait to receive the program [5]. Computer generated randomisation (Version 14.1, StataCorp, College Station, TX) was conducted by a researcher who had not had any contact with the families.

### Intervention

**Fig. 1:**
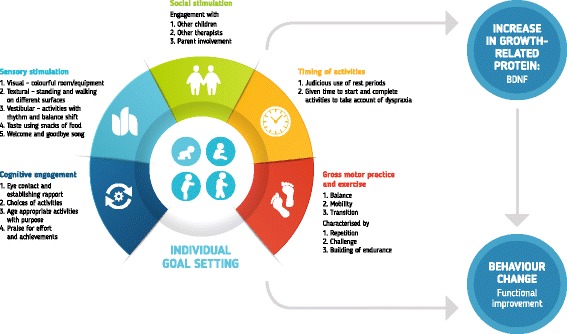
Schematic diagram showing the components of the enriched environment intervention and hypothesised effects of increased BDNF levels and improved functional abilities

The intervention provided a rich sensori-motor environment and included multiple supported activities selected to target individual goals in the development of motor skills and endurance. Activities focused particularly on balance and walking and included a high volume of practice aiming to increase BDNF production [22]. The intervention was also consistent with Motor Learning Theory [23], including opportunities for practice, intrinsic and extrinsic feedback, judicious use of rest periods, and performance of tasks in a variety of conditions that provided choice [23]. Each motor activity was supplemented with visual (eg, toys, applications), auditory (eg, songs, praise), taste (eg, snacks of food), vestibular (eg, balance shift) and tactile (eg, walking on differently textured surfaces) stimulation to build the richness of the sensori-motor environment (Fig. 1). The intervention was conducted for 2 to 3 h on six mornings per week with one-on-one supervision provided by a physiotherapist at RSCRI. Each therapist-child dyad worked individually and in small groups to provide additional social stimulation.

### Outcomes

The primary outcome was gross motor skill measured using the Rett Syndrome Gross Motor Scale (RSGMS) [21], comprising 15 items for sitting, standing, walking and transition activities rated according to the observed level of assistance (no assistance, mild, moderate or maximal assistance/unable) and using a 0 to 3 scale with 3 representing better function. The scale has demonstrated strong internal consistency, expected relationships between scores, age and genotype, excellent test-retest reliability and an observed difference of four points in an individual would represent change beyond within-subject error [21]. Gross motor skills were videotaped at RSCRI and a blinded assessor coded the gross motor skills. Gross motor data were collected at the beginning and end of the baseline period and at 2-monthly intervals during the intervention period.

The secondary outcomes were blood levels of the BDNF protein, growth, sleep quality and behaviour. Blood samples were taken at the beginning and end of the baseline period and then again after the six-month intervention period. For each test, blood was taken in the afternoon after lunch to standardise the time of day and relation with food intake [24]. Samples were collected in anticoagulant tubes and stored at −80 degrees C. At the time of testing, the whole blood was lysed with 3% triton X-100 (Amresco, Ohio) with sonification for 5 s for 20 times with a 10-s interval between each disruption (Scientz98-III, Scientz Biotechnology Co Ltd., Ningbo) [25]. Disrupted cell membranes were discarded by centrifuging the samples at 2000 g for 15 min at 4 °C. The supernatant was aliquoted for storage and the BDNF protein concentration was measured with commercially available two-site sandwich enzyme-linked immunoabsorbent assay (ELISA) kits (RAYBIOTECH, Norcross GA US; catalogue number ELH-BDNF Lot: 0520160106). Each blood sample was diluted *100 and tested in triplicate according to the manufacturer’s instructions. Mean values of the three concentrations of BDNF (ng/ml) were used for analysis.

The other secondary outcomes were measured at each of the assessment occasions including weight (kg) and supine height (cm) and BMI was derived. Sleep quality was measured using the disorders of initiating and maintaining sleep (DIMS) subscale of the parent-reported Sleep Disturbance Scale for Children (SDSC), comprising seven items rated on a five-point Likert scale with higher scores indicating more difficulties [26]. Sleep hygiene was measured with the Sleep Habits Questionnaire [27] as an adjusting variable because of potential effects on sleep quality [28]. Mood was measured using the mood subscale of the parent-completed Rett Syndrome Behaviour Questionnaire (RSBQ), with eight items rated on a 0 to 2 scale with 2 representing greater behavior problems [29].

Previously collected data reported to the InterRett database included the results of genetic testing for the type of *MECP2* mutation, age of developmental regression and the presence of epilepsy. A portion of one of the blood samples was used for Sanger sequencing for genotyping of the *BDNF* gene (SNP rs6265) to test the type of *BDNF* polymorphism (Val/Val, Val/Met, Met/Met).

The trial was registered with the Australian New Zealand Clinical Trials Registry (ACTRN12615001286538). Ethical approval was provided by the Human Research Ethics Committees at University of Western Australia, Perth (RA/4/1/7782) and the Shenzhen Children’s Hospital, Shenzhen (2015 [014]) and all families provided informed consent prior to participation according to the Declaration of Helsinki.

### Analysis

Based on previous data [5], we estimated a sample size of 12 girls would give us 80% power to detect a within-subject improvement in RSGMS scores of at least 4 points using a 5% two-tailed test. Descriptive statistics were used to summarise the baseline characteristics of the participants. Mean (SD) and median (range) values of the continuous outcome variables (RSGMS score, blood BDNF level, BMI, DIMS subscale score, RSBQ mood subscale score) at each time point and for each *BDNF* polymorphism were presented. A subset of RSGMS item scores were missing and they were imputed using last observation carried forward method. Intra-class correlation coefficients (ICC) (using two-way mixed effects models) and their 95% confidence intervals (CI) were employed to estimate the within-subject consistency in values of outcome variables during the baseline period (i.e. between time point 1 and 2).

Linear mixed-effects regression models with random intercepts were used to investigate the effects of treatment on the outcome variables. Time points 1 and 2 were free of intervention and thus the treatment variable was coded 0. For time points 3, 4 and 5 the treatment variable was coded 1, 2, and 3 respectively as it was hypothesised that there would be a dose-response relationship between intervention and outcome. Age at assessment was used as the time variable in the model. Univariable, as well as multivariable models, were fitted. In addition to age at regression, other potential confounders including blood BDNF level and the Malow sleep routines score [27] were used in the multivariable analyses where appropriate. Because blood BDNF level at time point 5 was not available for 2 participants, these individuals were removed from modelling involving blood BDNF level. Crude and adjusted estimates and their 95% confidence intervals were reported. All data analyses and data management were undertaken using Stata version 14.2.

## Results

The study was discussed with 13 families registered with InterRett of whom 12 (92.3%) provided informed consent. The mean (SD) age of the children at the first baseline assessment was 3.0 (1.1) years ranging from 1.5 to 5.2 years. Their mean (SD) age at regression had been 1.5 (0.4) years ranging from 1 to 2.3 years. Each had a pathogenic *MECP2* mutation and most of the common mutation categories were represented (C-terminal [*n* = 1], Early truncating [*n* = 1], Large deletion [*n* = 1], p.Arg168* [*n* = 1], p.Arg255* [*n* = 2], p.Arg270* [*n* = 3], p.Arg294* [*n* = 1] and p.Thr158Met [*n* = 2]). The *BDNF* polymorphism was homozygous (Val/Val) in three girls, heterozygous (Val/Met) in seven and negative (Met/Met) in two girls. At the first baseline assessment, five girls walked independently, four walked with assistance and three were unable to walk. One girl aged 18 months (p.Arg255*, Val/Met *BDNF* polymorphism) had been diagnosed with epilepsy prior to the commencement of the study which was managed with Levitircetam, and two girls developed epilepsy during the intervention period and commenced Valproate.

Mean (SD) values for each of the outcomes at each of the assessments are shown in Table 1. The RSGMS score was similar at baseline assessments 1 and 2, whether the duration of baseline was 1 month (ICC 0.977 [CI 0.751, 0.998], *p* = 0.003), two (ICC 0.987 [CI 0.813, 0.999], *p* = 0.001) or 3 months (ICC 0.976 [CI 0.772, 0.998], *p* = 0.003). The baseline blood BDNF levels were less variable when the duration of baseline was 1 month (ICC 0.615 [CI -0.31, 0.968], *p* = 0.120) than for 2 months (ICC 0.223 [CI -0.957, 0.969], *p* = 0.392) and 3 months (ICC 0.309 [CI -1.042, 0.939], *p* = 0.327), but the average differences were small at 27.7 ng/ml, 40.6 ng/ml and 9.9 ng/ml respectively. In total, video for 116/900 gross motor items were missing over the five testing occasions equating to 2 of 15 items per assessment per participant. Most missing items were the most complex skills and the previously observed level was carried forward as a conservative estimate. Because of illness (one each with lower respiratory tract infection and seizures), two gross motor assessments and post intervention blood tests could not be measured.

**Table 1:** Mean (SD) values for the primary and secondary outcomes at each assessment during the baseline and intervention periods

Outcome	Baseline period				Intervention period					
	Assessment 1		Assessment 2		Assessment 3		Assessment 4		Assessment 5	
	*N*	Mean (SD)	*N*	Mean (SD)	*N*	Mean (SD)	*N*	Mean (SD)	*N*	Mean (SD)
Rett Syndrome Gross Motor Scale (/45)	12	22.7 (9.6)	12	22.4 (10.4)	12	25.5 (8.9)	12	27.6 (8.4)	12	29.8 (9.7)
Blood Brain Derived Neurotrophic Factor (ng/ml)	11	165.0 (28.8)	12	146.1 (50.1)	–	–	–	–	10	510.0 (104.2)
BMI	12	15.2 (1.4)	12	15.1 (1.5)	12	14.6 (1.0)	11	14.7 (1.5)	11	15.0 (1.6)
Sleep Disturbance Scale for Children (DIMS subscale, /35)*	12	15.8 (5.5)	12	16 (6.1)	12	15.6 (4.3)	11	15.4 (7.2)	11	14.4 (5.6)
Rett Syndrome Behaviour Questionnaire (Mood subscale, /16)*	12	8.6 (3.6)	12	7 (3.6)	12	7.6 (3.6)	11	7.3 (3.0)	11	8.7 (3.5)

*One child unwell at assessment 4 and another at assessment 5

Compared to baseline, the within-person RSGMS score increased by 3.4 (95%CI 1.5, 5.3) points after 2 months of treatment, 5.7 (95%CI 3.3, 8.2) after 4 months, and 8.2 (95%CI 5.1, 11.2) after 6 months of treatment, adjusting for age of regression and current age. RSGMS scores increased 1.1 (95%CI 0.1, 2.2) point per additional month in age at the time of regression. For the 10 girls with post intervention BDNF data, there was a 10.3 (95%CI 3.2, 17.4) point increase in RSGMS score after 6 months of treatment adjusting for the effects of age at regression, current age and BDNF levels. Compared to baseline, BDNF levels increased by 321.4 ng/ml (95%CI 272.0, 370.8) after 6 months of treatment, adjusting for age at regression, current age and RSGMS score. BDNF levels increased an additional 39.0 ng/ml (95%CI 15.2, 62.4) for each additional year in age, adjusting for age at regression and the RSGMS score (Table 2).

**Table 2:** Univariable and multivariable models showing relationships between intervention, Rett Syndrome Gross Motor Scale (RSGMS) scores (*n* = 12) and blood brain derived neurotrophic factor (BDNF) levels (*n* = 10) adjusting for relevant confounders

Outcomes	Coefficient (95%CI)	*P* value	Coefficient (95%CI)	*P* value	Coefficient (95%CI)	*P* value
	Univariable model (*n* = 12)		Multivariable model – 1 (*n* = 12)		Multivariable model – 2 (*n* = 10)	
RSGMS						
Treatment after 2 months	2.7 (0.8, 4.6)	0.01	3.4 (1.5, 5.3)	0.001		
Treatment after 4 months	4.6 (2.2, 7.0)	< 0.001	5.7 (3.3, 8.2)	< 0.001		
Treatment after 6 months	6.6 (3.7, 9.5)	< 0.001	8.2 (5.1, 11.2)	< 0.001	10.29 (3.20, 17.38)	0.004
Age (year)	1.0 (−3.1, 5.2)	0.63	−1.6 (−6.0, 2.9)	0.492	−0.12 (−4.70, 4.47)	0.960
Age at regression (month)			1.1 (0.1, 2.2)	0.028	1.06 (0.07, 2.05)	0.035
Blood BDNF (time-varying)					−0.01 (−0.03, 0.01)	0.412
	Univariable model (*n* = 10)		Multivariable model (*n* = 10)			
Blood BDNF						
Treatment after 6 months	342.9 (297.0, 388.8)	< 0.001	321.4 (272.0, 370.8)	< 0.001		
Age (year)	30.1 (10.7, 49.5)	0.002	39.0 (15.6, 62.4)	0.001		
Age at regression (month)			−4.8 (−10.4, 0.9)	0.098		
RSGMS (time-varying)			2.4 (−0.6, 5.4)	0.119		

Adjusting for covariates, BMI decreased slightly after 2 months of treatment (−0.6 kg/m^2^ [−1.2. -0.1]) but thereafter increased and after 6 months of treatment was similar to baseline values (−0.3 kg/m^2^ [−1.2, 0.6]); the DIMS subscale scores remained similar to baseline values throughout the treatment period as did the mood subscale of the RSBQ (Table 3).

**Table 3:** Univariable and multivariable findings for relationships between treatment, BMI and other secondary behavioural outcomes (*n* = 12)

Outcomes	Univariable model		Multivariable model	
	Coefficient (95%CI)	*P* value	Coefficient (95%CI)	*P* value
BMI				
Treatment after 2 months	−0.5 (−1, 0.0)	0.042	−0.6 (−1.2, −0.1)	0.026
Treatment after 4 months	−0.4 (−1.0, 0.2)	0.157	−0.6 (−1.3, 0.1)	0.096
Treatment after 6 months	0.0 (−0.6, 0.6)	0.938	−0.3 (−1.2, 0.6)	0.49
Age (years)	−0.3 (−0.9, 0.3)	0.333	−0.3 (−1.1, 0.5)	0.519
Age at regression (months)			0.0 (−0.2, 0.2)	0.707
Gross motor assessment score (time-varying)			0.0 (0.0, 0.1)	0.307
Sleep Disturbance Scale for Children - DIMS subscale				
Treatment after 2 months	0.4 (−1.8, 2.6)	0.73	0.2 (−2.1, 2.4)	0.892
Treatment after 4 months	0.3 (−2.1, 2.7)	0.81	0.1 (−2.4, 2.6)	0.961
Treatment after 6 months	0.1 (−2.4, 2.6)	0.93	0 (−2.8, 2.8)	0.998
Age (years)	−2.6 (−4.7, −0.6)	0.01	−2.2 (−4.8, 0.3)	0.082
Age at regression (months)			−0.1 (−0.7, 0.4)	0.6
Sleep hygiene score			−0.1 (−0.3, 0.2)	0.567
Rett Syndrome Behaviour Questionnaire – Mood subscale				
Treatment after 2 months	−0.1 (−1.8, 1.6)	0.921	−0.2 (−2.0, 1.5)	0.804
Treatment after 4 months	−0.2 (−2.0, 1.7)	0.874	−0.4 (−2.3, 1.5)	0.691
Treatment after 6 months	1 (−0.9, 2.9)	0.31	0.7 (−1.3, 2.7)	0.496
Age (years)	−0.8 (−2.1, 0.6)	0.275	−0.2 (−1.7, 1.4)	0.789
Age at regression (months)			−0.2 (−0.5, 0.2)	0.316

The median (range) RSGMS and blood BDNF levels at each assessment are presented in Table 4 for each of the BDNF polymorphisms. Girls with the heterozygous polymorphism (Val/Met) had the lowest median RSGMS scores and blood BDNF levels at baseline but the magnitude of increase after 6 months of treatment was similar to girls with the Val/Val or Met/Met polymorphisms. The increase in score as a proportion of the baseline score was highest in girls with the Val/Met polymorphism (62%) compared with those with the Val/Val (29%) or Met/Met (32%) polymorphisms (Table 4).

**Table 4:** Median and range Rett Syndrome Gross Motor Scale (RSGMS) scores and blood brain derived neurotrophic factor (BDNF) levels at each assessment by BDNF gene polymorphism (Val/Val, Val/Met, Met/Met)

	Baseline period				Intervention period					
	Assessment 1		Assessment 2		Assessment 3	Assessment 4			Assessment 5	
RSGMS										
	*N*	Median (range)	*N*	Median (range)	*N*	Median (range)	*N*	Median (range)	*N*	Median (range)
Val/Val	3	31 (30–34)	3	32 (30–33)	3	32 (30–35)	3	34 (32–37)	3	39 (30–39)
Val/Met	7	16 (4–29)	7	16 (0–29)	7	23 (9–33)	7	24 (13–34)	7	26 (13–38)
Met/Met	2	28.5 (27–30)	2	28.5 (27–30)	2	31.5 (30–33)	2	33.5 (33–34)	2	37.5 (36–39)
Blood BDNF										
Val/Val	3	173.8 (152.1–177.9)	3	166.6 (126.7–214.2)	–	–	–	–	3	493.1 (436.3–689.0)
Val/Met	6^a^	156.3 (117.5–232.6)	7	129.5 (69.8–211.4)	–	–	–	–	6^a^	446.1 (387.1–619.6)
Met/Met	2	172.39 (166.9–177.9)	2	147.5 (82.1–212.9)	–	–	–	–	1^a^	566.7 (566.7–566.7)

^a^Missing blood sample at assessment 1 due to technical difficulties and missing blood samples at assessment 5 due to ill health

## Discussion

Motor learning and exercise supplemented with rich social, cognitive and other sensory experiences had positive effects on motor functioning in Rett syndrome. Adjusting for covariates, participants gained on average eight points on the 45-point motor scale after 6 months treatment, much greater in magnitude than the response observed in the conductive education study after 6 months [5]. Importantly, gross motor improvement was observed for children with different abilities at baseline and included achievement of independent sitting, walking or transition skills such as sitting to standing. We did find that children with a later age at regression, usually observed with *MECP2* mutations associated with milder clinical severity [30], gained slightly more gross motor skills over the treatment period. It is not known whether a longer treatment period was necessary to achieve greater gross motor gains for those who had experienced earlier regression.

General health issues were not compromised. Growth and sleep patterns were maintained over the study period and the intervention did not appear to affect mood, although the mood subscale of the RSBQ developed for Rett syndrome may not have been sensitive to changes in mood in preschool children. Two girls in the current study developed epilepsy during the intervention period which was not inconsistent with the natural history of Rett syndrome where 50% develop epilepsy by 5 years of age [31]. We observed that an environmental enrichment intervention could be safely delivered to girls with Rett syndrome.

To our knowledge, this is the first intervention study investigating serum BDNF levels as an outcome in a genetically characterised sample of children with Rett syndrome. Baseline BDNF levels in our sample were generally lower than in children in the general population measured using the same assay [32], similar to animal studies where BDNF protein levels in the brain of MecP2 mutant mice were reduced compared with wild-type levels [33]. In contrast, BDNF levels in children with autism spectrum disorder [34] and Down syndrome are high [35] compared with the general population. BDNF levels vary with factors such as the time of the day [24, 36] and age [36], and further standardized assessment in children with Rett syndrome is necessary to understand BDNF production in Rett syndrome and explain any variance between different neurodevelopmental disorders.

An important mechanism underpinning the beneficial effects of environmental enrichment is believed to be increased levels of BDNF, which promotes the survival and growth of neurones, synaptic efficiency and neuroplasticity [6]. In studies of MeCP2 knockout mice, strategies to improve BDNF signalling have included administration of IGF-1 [37] and the provision of an enriched environment [12, 38]. In 12 girls with a *MECP2* mutation, peripherally administered IGF-1 was associated with modest improvement in autonomic and behavioural features [39]. General population studies suggest that aerobic exercise is associated with increases in BDNF levels [40]. In our present study, the provision of an enriched and supported environment intervention that included exercise was associated with a threefold increase in serum BDNF levels alongside parallel gains in gross motor skills. Although we cannot discern the ingredients of the intervention that were associated with changes in BDNF levels, we recognize that administration of any exercise regimen for young children with movement deficits requires training and learning in an appealing environment that promotes, rewards and challenges activity.

We also included the polymorphism status of the *BDNF* gene in our analyses. The substitution of valine for methionine at codon 66 (Val66Met) is a common *BDNF* polymorphism which can impair intracellular trafficking and secretion of the resulting mature BDNF protein [33] and in *Mecp2* knockout mice, reduced dendritic growth and complexity and reduced frequency of postsynaptic currents were found [41]. In Rett syndrome, the presence of the Val/Met *BDNF* polymorphism has been associated with slightly greater clinical severity [7] consistent with the current study where girls with the Val/Met polymorphism had the poorest gross motor skills at baseline. Two of the three individuals with seizures had the Val/Met polymorphism and the other the Met/Met polymorphism. One of these Val/Met individuals had seizure onset prior to 2 years of age in contrast with another study there were no individual with this genotype experienced seizures prior to 2 years of age [8]. It would be reasonable to expect that not all individuals with Rett syndrome would benefit equally from increased BDNF because of the type of *MECP2* mutation or *BDNF* polymorphism. A six-week high-intensity exercise program resulted in cognitive benefits for children in the general population compared to an active control group, and the greatest improvements were observed in children who were Met carriers compared to those with homozygous *BDNF* status [42]. Similar to this recent literature, we also observed different responses to training with the change in gross motor scores as a proportion of baseline scores being greatest for girls with the Val/Met polymorphism. In contrast to previous studies of mainly Caucasian girls with Rett syndrome [7–9], more girls in the current sample had the Val/Met rather than the Val/Val polymorphism similar to other studies of Asian populations [43]. Investigations of larger sample sizes are needed to investigate the relationships between the *BDNF* polymorphism and responses to an enriched environment.

We did not conduct a conventional randomized controlled trial because of sample size availability and the striking variability in motor abilities between different genotypes, which limited our capacity to randomize into two similar groups. A small proportion of video data was missing and our sample size precluded longitudinal modelling to estimate the missing data. We therefore carried forward the last known value for each item for each child, consistent with our observations that no child lost skill over the course of the intervention and resulting in a conservative estimate of functioning. We also do not know whether increased blood levels of BDNF over the study period were reflected in brain tissues, but we are encouraged by the parallel increases in gross motor skills and evidence that serum BDNF can cross the blood-brain barrier in young animals [44]. We nevertheless implemented strategies to minimise bias. We recruited children with minimal exposure to interventions enabling us to more clearly identify an effect; we randomised individuals to the duration of the baseline period and then assessed under control and intervention conditions; we standardised the timing of blood draws to reduce diurnal variation in serum BDNF level [24]; and we used a blinded assessor to code the video data. We still captured variability in *MECP2* mutation and BDNF polymorphism types and different levels of baseline skills but recommend replication of our methods to accumulate more evidence.

## Conclusions

Environmental enrichment has long been applied as an intervention to ameliorate the effects of brain disorders on functioning. We suggest that the enriched environment has benefits for children with Rett syndrome and clinicians and therapists should plan therapy and activity opportunities accordingly. New drug, cell and gene therapies for neurological disorders including Rett syndrome are being developed that use BDNF pathways [45]. We propose that behavioural interventions such as environmental enrichment will be an important adjunct in the evaluation of these new therapies because environmental enrichment also has implications for functioning.

## Abbreviations

BDNF: Brain derived neurotrophic factor
DIMS: Disorders of initiating and maintaining sleep
ELISA: Enzyme-linked immunoabsorbent assay
ICC: Intra-class correlation coefficients
*MECP2*: Methyl-CpG-binding protein 2
RSBQ: Rett syndrome behaviour questionnaire
RSGMS: Rett syndrome gross motor scale
SDSC: Sleep disturbance scale for children
